# Supplementary material for: Lack of Neuroprotective Effects of High-Density Lipoprotein Therapy in Stroke under Acute Hyperglycemic Conditions
Source: Molecules. 2021 Oct 21;26(21):6365. doi: 10.3390/molecules26216365 (PMC8588473; doi:10.3390/molecules26216365)
Supplement: Supplementary file 1 [file molecules-26-06365-s001.zip › molecules-1366220-supplementary.pdf]

Supplementary Materials

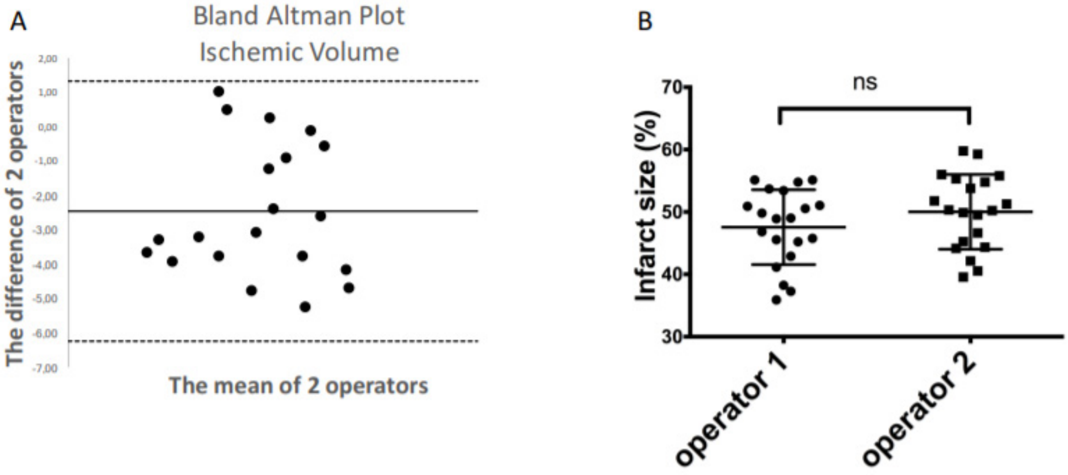

Figure S1. (**A**) Bland Altman Plot: the bias of measurement of the 2 operators was −2.46%. Upper limit of agreement (LOA): 1.33%. Lower LOA: −6.25%. There was a good correlation between the 2 operators for the analysis of the infarct volume. (**B**) Comparison of infarct size according to the assessment of the two operators. There was no statistical difference between the 2 operators regarding the assessment of infarct size. (47.56 ± 1.34% vs 50.02 ± 1.3% $p = 0.20$). Data are represented by means ± SD.
